# Editorial Comment on A case of mediastinal teratoma with malignant transformation into angiosarcoma and relapse with multiple bone metastases that was cured by multidisciplinary treatment

**DOI:** 10.1002/iju5.12564

**Published:** 2022-12-05

**Authors:** Takeshi Kishida

**Affiliations:** ^1^ Kanagawa Cancer Center Hospital Yokohama Kanagawa Japan

The metastatic germ cell tumor (GCT) is a highly curable disease with cisplatinum‐based multidrug regimen even for patients with multiple organ metastasis. However, teratoma malignant transformation (TMT), which sometimes occurs in the course of GCT treatment, is one of the difficult situations in this curable disease. Usually, TMT is chemo‐resistant, and the complete resection is the only way to cure it. For unresectable situations, chemotherapy targeted to GCT will be applied and histology‐driven chemotherapy is rare because many of them are chemo‐resistant. Giannatempo reported that in the 320 cases of TMT, 4.4% and 49.7% of patients received chemotherapy for the specific histology tailored and GCT tailored, respectively.^1^ On the other hand, the soft tissue sarcoma (STS) is also one of the chemo‐resistant malignancies. When unresectable, the STS is treated by anthracycline‐based chemotherapy irrespective of the histology. However, some specific types of sarcomas are effectively treated by specific regimens. For angiosarcoma, NCCN guidelines recommended taxane‐based chemotherapy.^2^, ^3^ Phase II study of weekly paclitaxel revealed 78% progression‐free after two cycles and 10% histological complete response in 30 cases of angiosarcoma.^2^

In the present report,^4^ the authors successfully treated the angiosarcoma of TMT. Their multidisciplinary method using operation, sensitive chemotherapy, and radiation therapy resulted in long‐term survival preserving the patient's QOL. Although TMT is extremely rare and the prognosis is usually poor, the present case gives us an encouraging strategy for the treatment of this difficult disease.

## Conflict of interest

The author declares no conflict of interest.
